# α-Secretase nonsense mutation (*ADAM10* Tyr167*) in familial Alzheimer’s disease

**DOI:** 10.1186/s13195-020-00708-0

**Published:** 2020-10-31

**Authors:** Pablo Agüero, María José Sainz, María-Salud García-Ayllón, Javier Sáez-Valero, Raquel Téllez, Rosa Guerrero-López, Julián Pérez-Pérez, Adriano Jiménez-Escrig, Estrella Gómez-Tortosa

**Affiliations:** 1 Department of Neurology, Fundación Jiménez Díaz, Avenida de los Reyes Católicos 2, 28040 Madrid, Spain; 2 Instituto de Neurociencias de Alicante, Universidad Miguel Hernández-CSIC, Sant Joan d’Alacant, Spain; 3 Centro de Investigación Biomédica en Red sobre Enfermedades Neurodegenerativas (CIBERNED), Sant Joan d’Alacant, Spain; 4 Unidad de Investigación, Hospital General Universitario de Elche, Fundación para el Fomento de la Investigación Sanitaria y Biomédica de la Comunitat Valenciana (FISABIO), Elche, Spain; 5 Department of Immunology, Fundación Jiménez Díaz, Madrid, Spain; 6 Instituto de Investigación Sanitaria Fundación Jiménez Díaz (IIS-FJD) and CIBERER, Madrid, Spain; 7 Secugen S.L., Madrid, Spain; 8 Department of Neurology, Hospital Ramón y Cajal, Madrid, Spain

**Keywords:** ADAM10, α-Secretase, Familial Alzheimer’s disease, Genetics

## Abstract

**Background:**

The disintegrin metalloproteinase 10 (ADAM10) is the main α-secretase acting in the non-amyloidogenic processing of APP. Some *ADAM10* gene variants have been associated with higher susceptibility to develop late-onset AD, though clear clinical-genetic correlates remain elusive.

**Methods:**

Clinical-genetic and biomarker study of a first family with early- and late-onset AD associated with a nonsense *ADAM10* mutation (p.Tyr167*). CSF analysis included AD core biomarkers, as well as Western blot of ADAM10 species and sAPPα and sAPPβ peptides. We evaluate variant’s pathogenicity, pattern of segregation, and further screened for the p.Tyr167* mutation in 197 familial AD cases from the same cohort, 200 controls from the same background, and 274 AD cases from an independent Spanish cohort.

**Results:**

The mutation was absent from public databases and segregated with the disease. CSF Aβ42, total tau, and phosphorylated tau of affected siblings were consistent with AD. The predicted haploinsufficiency effect of the nonsense mutation was supported by (a) ADAM10 isoforms in CSF decreased around 50% and (b) 70% reduction of CSF sAPPα peptide, both compared to controls, while sAPPβ levels remained unchanged. Interestingly, sporadic AD cases had a similar decrease in CSF ADAM10 levels to that of mutants, though their sAPPα and sAPPβ levels resembled those of controls. Therefore, a decreased sAPPα/sAPPβ ratio was an exclusive feature of mutant *ADAM10* siblings. The p.Tyr167* mutation was not found in any of the other AD cases or controls screened.

**Conclusions:**

This family illustrates the role of *ADAM10* in the amyloidogenic process and the clinical development of the disease. Similarities between clinical and biomarker findings suggest that this family could represent a genetic model for sporadic late-onset AD due to age-related downregulation of α-secretase. This report encourages future research on ADAM10 enhancers.

## Introduction

Since mutations in the *PSEN1*, *PSEN2*, and *APP* genes were reported as causes of autosomal dominant early-onset familial Alzheimer’s disease (EOAD), no other direct genetic causes have been associated with AD. In late-onset AD (LOAD), presence of the ε4 variant in the *APOE* genotype increases the risk of developing the disease by 4-fold (one allele) to > 10-fold (two alleles) [1]. In addition, the latest advances in AD genetics have further identified more than 45 genes/loci associated with increased risk of developing AD [2], although to date no mutations have been reported to directly cause LOAD.

All known genetic causes of EOAD are related to the same pathogenic process, that is, abnormal processing of the amyloid precursor protein (APP) and subsequent pathological accumulation of Aβ peptides in the brain [3]. In the non-amyloidogenic processing of APP, the protein is proteolyzed through the α-secretase pathway, resulting in soluble fragments (sAPPα) formation [4]. Several enzymes in the disintegrin and metalloprotease (ADAM) family have α-secretase activity in vitro. Particularly, ADAM10 has been identified as the major α-secretase responsible for ectodomain shedding of APP in the brain [5–7]. ADAM10 is a transmembrane and secreted protein of 748 amino acids in length that plays a role in cell adhesion and proteolytic processing of the ectodomains of more than 40 substrates, several of which are crucial for normal brain development and function [7–9].

The role of ADAM10 in the non-amyloidogenic processing of APP, shown in vitro and in animal models [7–10], has pointed to *ADAM10* as a top candidate gene for involvement in the pathophysiology of AD [11]. However, a clear clinical-genetic correlation of α-secretase haploinsufficiency has not been reported to date. Only some *ADAM10* variants have been associated with higher susceptibility to LOAD, and two rare *ADAM10* missense mutations have been observed in a small number of LOAD kindreds, though segregation was incomplete [12].

Here, we present a family with AD (with both early and late onset) associated with a heterozygous nonsense mutation in *ADAM10* (p.Tyr167*). CSF biomarkers of two affected patients were consistent with α-secretase haploinsufficiency. Evidence from this family further implicates the amyloidogenic process and the role of α-secretase activity in the development of AD. Some similarities between clinical and biomarker findings suggest that this family could represent a genetic model of sporadic late-onset AD due to an age-related down-regulation of α-secretase.

## Methods

### Participants

**Fig. 1:**
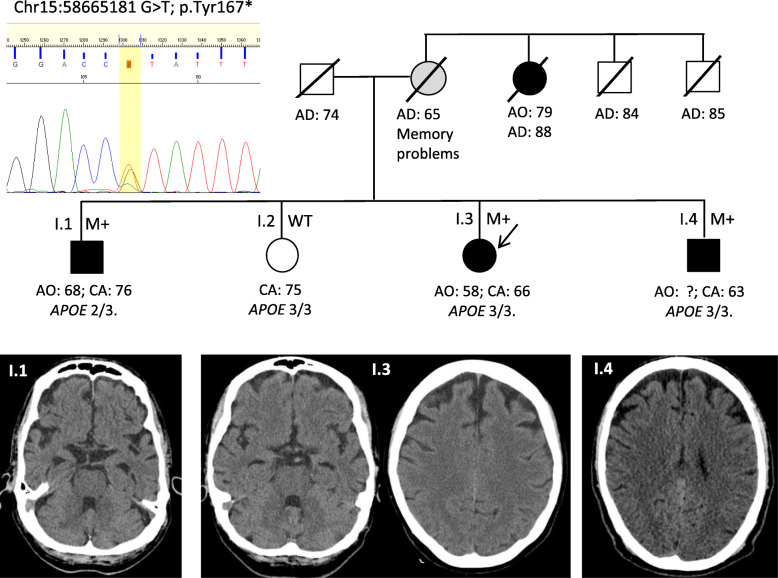
Family tree including *ADAM10* Tyr167*mutation and clinical status. CT scan images of the three affected siblings showing patterns of atrophy. Black M+, affected carrier; white WT, non-affected wild-type; gray, possibly affected. Age at onset (AO), at death (AD), or current age (CA) in years. Chromatogram of the mutation is also shown

This family (Fig. 1) belongs to a cohort of cases with familial dementia of the Alzheimer type (DAT) recruited from the memory clinic of Fundación Jiménez Díaz (Madrid, Spain). The family comprises a proband case (I.3) with early-onset AD, two affected brothers, (I.1 and I.4), and an elderly healthy sister (I.2)*.* The family history was given a Goldman score of 2 (familial aggregation of three affected members) [13]. Suspected inheritance was through the maternal side, as the mother had died at age 65 years with mild memory problems, and her sister had developed LOAD in her late 70s. Study of the family was approved by the Research Ethics Committee at Fundación Jiménez Díaz, and informed consent for biological marker and genetic studies was provided by patients or surrogates.

### Procedures

#### Genetic study

DNA was extracted from peripheral blood leukocytes using the QIAamp DNA blood Mini-kit (Qiagen). The genetic study of the proband (I.3) included a NGS panel with the coding region of the following genes: *PSEN1*, *PSEN2*, *APP*, *MAPT*, *PGRN*, *VCP*, *CHMP2B*, *TARDBP*, *FUS*, *ADAM10*, *SORL1*, *SNCA*, *TREM2*, *UBIQLN2*, *ITM2B*, *CSF1R*, *TYPOBP*, and *SQSTM1* (amplified by Ampliseq technology and sequenced using a Miseq equipment, Illumina, yielding a coverage of over 200x). The *APOE* genotype and the hexanucleotide expansion in intron 1 of *C9ORF72* were also analyzed. Potential pathogenic variants were reviewed in genomic databases (dbSNP, ExAc, Genome Aggregation Database, and Exome Variant Server) and analyzed for pathogenicity with prediction programs. Potentially pathogenic variants in the proband and siblings were confirmed by Sanger sequencing (Big Dye v3.1; ABI 3730, Applied Biosystems). The segregation pattern was analyzed in the family. After identifying the p.Tyr167* mutation, we further screened for this particular *ADAM10* variant in another 197 familial DAT samples from the same cohort, 200 controls from the same population background, and 274 AD cases from an independent Spanish cohort, provided by the Banco Nacional de ADN Carlos III (Salamanca University).

#### CSF analyses

CSF samples from cases I.1 and 1.3 had been stored at − 80 °C. We conducted three types of analyses in CSF. First, we analyzed core AD biomarkers Aβ42, total tau (T-tau), and phosphorylated tau (P-tau) levels using Lumipulse G600II chemiluminescent immunoassay (Fujirebio Iberia, Barcelona, Spain), following the standardized commercial protocol. Cut-off points used to distinguish values consistent with AD were Aβ42 < 770 pg/ml, T-tau > 440 pg/ml, and P-tau > 58 pg/ml, and Aβ42/40 ratio < 0.068. Based on these values, we applied the Erlangen Score algorithm, which rates 0 to 4 [14].

Secondly, we speculated as to whether *ADAM10* haploinsufficiency would be evidenced at the protein level in CSF, and thus compared samples of p.Tyr167* carriers (cases I.1 and I.3) with sporadic age-matched AD cases with positive CSF AD biomarkers (*n* = 10), and age-matched controls (*n* = 12). We recently reported that ADAM10 is present in human CSF as three distinct species: a truncated soluble form lacking the intracellular C-terminal domain probably released from the membrane (sADAM10; ~ 50 kDa), a mature unprocessed full-length form (ADAM10f; ~ 55 kDa), and an immature full-length form retaining the prodomain (proADAM10; ~ 80 kDa) [15].

Third, we analyzed sAPPα and sAPPβ levels of the same samples (2 mutant cases, 10 sporadic AD cases, and 12 controls) to assess the biological effect of the ADAM10 mutation in APP processing. We previously showed that sAPP species associate in heteromeric complexes that may reduce the accuracy of ELISA [16]. For this reason, we used a sodium dodecyl sulfate-polyacrylamide gel electrophoresis (SDS-PAGE)-based approach to study these proteins instead of ELISA.

The 24 CSF samples were analyzed by western blotting as previously described [15, 17]. In brief, samples were denatured at 98 °C for 5 min and resolved by SDS-PAGE under reducing conditions. Following electrophoresis, proteins were blotted onto nitrocellulose membranes (Bio-Rad Laboratories GmbH, Munich, Germany) and probed with an anti-ADAM10 antibody that is specific for the mid-region, thus common to full-length and cleaved species (rabbit polyclonal; OAGA02442, Aviva Systems Biology, San Diego, USA). Samples were also blotted with an anti-sAPPα specific to the C-terminus of sAPPα (mouse monoclonal; IBL International, Hamburg, Germany) and an anti-sAPPβ specific to the C-terminus of sAPPβ (rabbit polyclonal; IBL, Hamburg, Germany). The specificity of these pan-specific sAPPα and sAPPβ antibodies had been tested for western blotting in a previous study [17]. Blots were then incubated with an IRDye 800 CW anti-rabbit secondary antibody (ADAM10; sAPPβ) or an IRDye 680RD anti-mouse antibody (sAPPα) (both from LI-COR Biosciences, Lincoln, NE, USA) and imaged on an Odyssey Clx Infrared Imaging System (LI-COR). Band intensities were analyzed using LI-COR software (Image Studio v 5.2.5). All samples were analyzed in duplicate. The immunoreactive ADAM10 signal for each band was normalized to the immunoreactivity of the corresponding band from a CSF sample (aliquots from the same sample), resolved in all blots. Statistical comparison was conducted using the Student *t* test.

## Results

### Clinical assessment

The family members have been evaluated over the past year (Table 1 and Fig. 1). Their ages at onset ranged from 58 to 68 years, though the LOAD presented by the maternal aunt, starting in her late seventies, could also be related to a p.Tyr167* mutation carrier status. The clinical features of the siblings are characterized by insidious onset and slow evolution.

**Table 1:** Summary of clinical and biomarker data. Evaluation of the proband case and the affected sibling conducted over the last year (current age)

	Case I.3 (proband, woman)	Case I.1 (brother)
**Age at onset**	58 years	68 years
**Symptoms at onset**	1st delusions, 2nd memory	Memory
**Current age**	66 years	76 years
**Neuropsychology**		
MMSE	16/30	23/30
Verbal fluency (1 min)	animals: 2; letter S: 2	animals: 7; letter S: 4
Boston Naming (15 items)	3/15 + 5 with phonetic clues	6/15 + 5 with phonetic clues
HVLT (12 items)	learning trials: 0-0-0 free recall: 0, cued: 0 recognition: 0	learning trials: 0-1-2 free recall: 0, cued: 0 recognition: 0
Clock Test	order 4/10, copy 6/10	order 2/10, copy 8/10
**Functional status**	GDS 5/CDR 2	GDS 3–4/CDR 1
CSF biomarkers (pg/ml) *		
Aβ42	635	708
Aβ 40	11,391	14,403
Ratio Aβ42/40	0.056	0.049
T-tau	960	1037
P-tau	176	184
Erlangen score	4 (probable AD)	4 (probable AD)
ADAM10 55 kDa **	- 39%	- 69%
ADAM10 50 kDa **	- 34%	- 54%
ADAM10 80 kDa **	- 47%	- 26%
sAPPα 110 kDa^#^	- 73%	- 66%
sAPPα 120 kDa^#^	- 70%	- 63%
**APOE genotype**	3/3	2/3
**Pattern of atrophy**	Frontal + hippocampal	Diffuse cortical + hippocampal

*CDR* Clinical Dementia Rating scale (0 to 3), *GDS* Global Deterioration Scale (0 to 7), *MMSE* Mini-Mental State Examination, *HVLT* Hopkins Verbal Learning Test

*Cut-off points to consider the values consistent with AD are as follows: Aβ42 < 770 pg/ml; T-tau > 440 pg/ml; P-tau > 58 pg/ml; ratio Aβ42/40 < 0.068

**Fig. 2:**
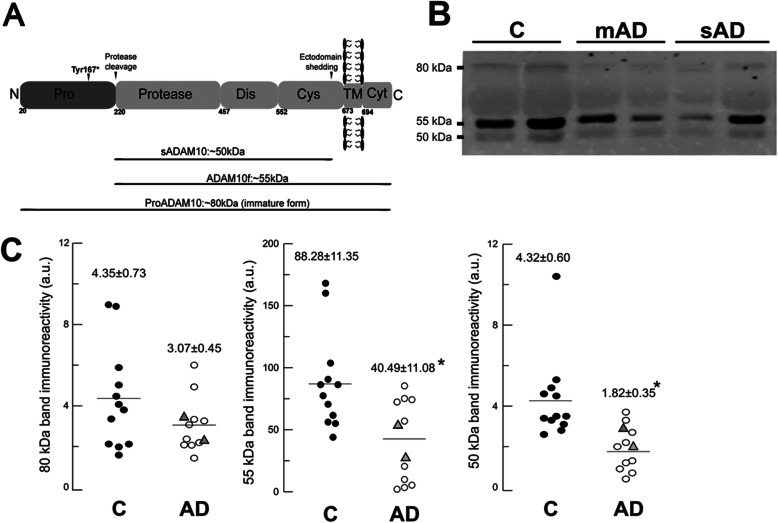
Levels of ADAM10 in AD CSF samples. **a** Schematic representation of the domains of transmembrane type I ADAM10 protein. **b** Western blots of CSF samples from controls (C), p.Tyr167* mutation carriers (mAD; cases I.1 and I.3), and sporadic AD (sAD) subjects using an ADAM10 antibody specific for the mid-region, common to all species. **c** Densitometric quantification of the 80-, 55-, and 50-kDa bands for the individual controls (*n* = 12; black circles) and AD cases (sAD, *n* = 10; white circles; mAD, *n* = 2; gray triangles). The data represent the means ± SEM in arbitrary units. For AD, means ± SEM represent values for sAD. *Significantly different (*p* < 0.007) from the control group, as assessed by Student’s *t* test (non-significant *p* = 0.17 value for the 80-kDa ADAM10 form)

** % Reduction of ADAM10 isoforms versus the mean for controls (shown in Fig. 2)

**Fig. 3:**
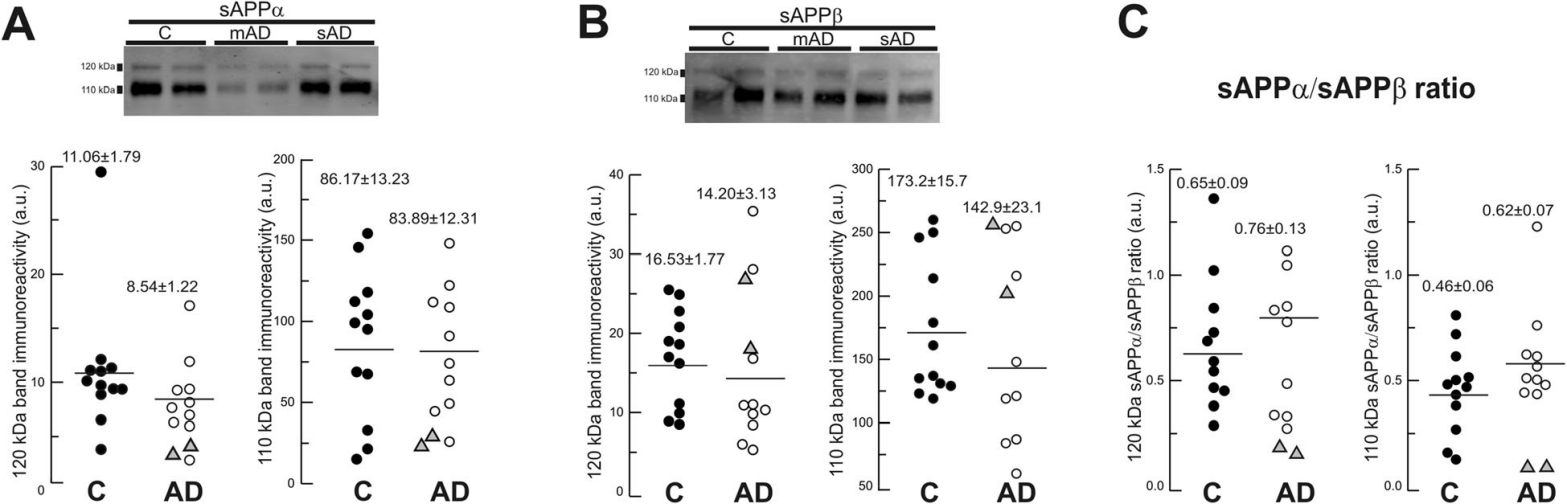
Levels of sAPPα and sAPPβ in CSF samples. Western blots of CSF samples from two controls (C), p.Tyr167* mutation carriers (mAD; cases I.1 and I.3), and sporadic AD (sAD) blotted with specific sAPPα (**a**) and sAPPβ (**b**) antibodies. Densitometric quantification of the 110- and 120-kDa sAPPα (**a**) and sAPPβ (**b**) bands for controls (*n* = 12; black circles) and AD cases (sAD, *n* = 10; white circles; mAD, *n* = 2; gray triangles). **c** Graphs of the sAPPα/sAPPβ ratios (for the 110- and 120-kDa species) for each sample. All data represent the means ± SEM in arbitrary units. For AD, means ± SEM represent values for sAD

^#  ^% Reduction of sAPPα isoforms, compared to controls (Fig. 3)

Case I.1 has an amnestic syndrome, and after 8 years with memory complaints, his MMSE is 23/30 and he is still able to conduct some outside-of-home chores independently. The initial symptoms of case I.3 consisted of persecutory delusions (being spied on at home) and delusional jealousy regarding her husband, followed after 2 years by progressive cognitive impairment. She is currently in a moderate stage of dementia. Functionally, she requires supervision for all activities of daily living, though she can collaborate in simple tasks, recognizes relatives and friends, and communicates verbally. She has not developed parkinsonism or seizures. As regards case 1.4, the age at onset of symptoms is unclear. There is evidence that he shared the persecutory delusions of his sister (I.3) while living at her home 5 years ago. Currently, he presents incipient dementia with mild cognitive and functional impairment (CDR = 0.5, GDS = 2). Screening tests have revealed impaired recent memory, but he has refused to undergo complete neuropsychological and CSF studies.

CT scan images of the three siblings are shown in Fig. 1, revealing predominance of frontal atrophy in siblings I.3 and I.4, while in case I.1 cortical atrophy is diffuse and mesial temporal atrophy is more pronounced.

The 75-year-old sister (I.2) was functionally independent for activities of daily living. She underwent a cognitive assessment that found results within normal limits.

### Genetic studies

The NGS dementia panel of case I.3 revealed a heterozygous nonsense mutation/stop codon c.501G>T: p.Tyr167* in *ADAM10* (genomic mutation coordinates according to GRch38.p13: chr15:58665181; protein nomenclature NP_001101.1; p.Y167*), which was confirmed by Sanger sequencing (chromatogram in Fig. 1). There were no other potentially pathogenic variants in the other genes included in the panel. The *C9ORF72* expansion was also within normal range, and the *APOE* genotype was 3/3. The *ADAM10* nonsense mutation was absent from the dbSNP, ExAc, Genome Aggregation Databases, and Exome Variant Server, and its predicted pathogenicity according to CADD score was 35 (> 20 suggests pathogenicity). A nonsense mutation generates a premature termination codon, the mutant mRNA transcript is likely degraded by nonsense-mediated mRNA decay, and the truncated protein is likely never formed.

The two affected brothers also carried the p.Tyr167* variant which was absent in the healthy 75-year-old sister (I.2) (Fig. 1), consistent with trait segregation*.* According to the guidelines of the American College of Medical Genetics and Genomics [18], the p.Tyr167* variant fulfilled all criteria to be considered “likely pathogenic”, as this variant is only found in cases and not in controls, segregates with disease, is predicted by two in silico programs to affect protein, and changes the protein-length or is a loss-of-function mutation. The mutation was not present in any of the other 471 DAT samples analyzed, nor in the population-background matched controls. We also failed to find the mutation in the Collaborative Spanish Variant Server (csvs.babelomics.org) which includes normal individuals as well as AD cases and patients with other diseases.

### CSF analyses

CSF levels of AD biomarkers showed elevated T-tau and P-tau and low levels of Aβ42 in the two affected siblings (Table 1), consistent with AD as indicated by NIA-AA Research Framework criteria A+T+N+ [19], and an Erlangen score of 4.

Regarding ADAM10 immunoreactivities in CSF, all samples revealed three immunoreactive species with apparent molecular masses of ~ 80 kDa, 55 kDa, and 50 kDa attributed respectively to immature (proADAM10), mature full-length (ADAM10f) and truncated soluble (sADAM10) species (as previously characterized in Sogorb-Esteve et al. [15]). We found that the 55-kDa ADAM10f species was significantly decreased in both mutation carriers compared to controls (Fig. 2): a decrease of 39% in case I.3 and of 69% in case I.1. The 50-kDa truncated sADAM10 also showed a 34% reduction in case I.3, and a 54% reduction in case I.1, as compared to controls. Interestingly, the sporadic AD cases had a similar pattern to mutants of decrease of both species as compared to controls: a 54% mean reduction of the 55-kDa peptide (40 ± 11versus 88 ± 11, means±SEM, *p* < 0.01) and 58% mean reduction of the 50-kDa fragment (1.8 ± 0.3versus 4.3 ± 0.6, *p* < 0.005). The concentration of the 80-kDa immature proADAM10 had no significant differences between sporadic AD and controls (*p* = 0.17), but again the mutation carriers displayed lower levels for this species (47% redution in case I.3, and 26% reduction in case I.1).

The analysis of sAPPα and sAPPβ levels in CSF showed that ADAM10 mutants had significantly reduced levels of sAPPα, but unaltered sAPPβ as compared to both sporadic AD and control cases (Fig. 3). The decreases in sAPPα were substantial for both the major 110-kDa band (decrease of 73% in case I.3 and of 66% in case I.1) and the 120-kDa band (decrease of 70% in case I.3 and of 63% in case I.1) (Fig. 3). These data are consistent with a biological effect of the p.Tyr167* mutation in decreasing the non-amyloidogenic APP processing through the α-secretase pathway. There were no significant differences in sAPPα or sAPPβ levels between sporadic AD and control cases. Therefore, a sAPPα/sAPPβ ratio did not show differences between sporadic AD and control cases, but displayed a substantial decrease in both mutant siblings (Fig. 3).

## Discussion

This family with an *ADAM10* nonsense mutation illustrates the first clinical-genetic correlate linking α-secretase haploinsufficiency with familial EOAD and LOAD and further implicates the amyloidogenic process in the development of the disease. Certain clinical and biomarker findings suggest that this family could represent a genetic model for sporadic LOAD with an age-related downregulation of α-secretase activity.

In the affected siblings, AD diagnosis was based on the recent NIA-AA Research Framework criteria [19], including a cognitive impairment consistent with an Alzheimer’s clinical syndrome, together with CSF biomarkers of two siblings consistent with A+T+N+. The clinical features of the family members were far less aggressive than the dementia phenotypes caused by *PSEN1* mutations, and more closely resembled LOAD. Moreover, the LOAD presented by the maternal aunt, starting in her late seventies, could have been related to a p.Tyr167* mutation carrier *status*.

A variety of evidence supports the pathogenicity of this p.Tyr167* mutation and its association with the AD of the family members through an α-secretase haploinsufficiency mechanism. First, the crucial role of ADAM10 in the non-amyloidogenic processing of APP has long made *ADAM10* a top candidate gene for involvement in the pathophysiology of AD [2]. There is a direct mechanism by which coding errors in the gene could reduce α-secretase activity and promote amyloid deposition. Indeed, over-expression of ADAM10 in mouse models has been shown to halt Aβ production and subsequent aggregation [10], while downregulation of ADAM10—through increased levels of its inhibitor SFRP1 protein—anticipates the appearance of Alzheimer changes in an AD-like mouse model [20].

Second, the type of mutation (nonsense generating a premature termination codon) predicts a deleterious change in protein length. The mutant mRNA transcript is likely degraded by nonsense-mediated RNA decay and the truncated protein probably never forms. In silico prediction programs assigned this type of mutations the highest pathogenic prediction score. Furthermore, the p.Tyr167* mutation is a novel variant. It does not exist in public databases, has not been reported after large-scale screenings of sporadic AD cases [21], and was not found in any other cases within our familial DAT cohort. It is interesting that the p.Tyr167* mutation resides in the prodomain region of the protein (amino acids 20 to 219, Fig. 2), the same region where two rare missense mutations have been described as being highly penetrant for LOAD (Q170H and R181G) [12]. The pathogenicity of these mutations has been addressed in transgenic mice, and these data can very likely be applied to our three-amino acid-preceding stop mutation. Suh et al. [22] have shown that the Q170H and R181G variants impair the molecular chaperone function of the ADAM10 prodomain, attenuate the α-secretase activity of the protein, and shift APP processing toward β-secretase-mediated cleavage, enhancing Aβ plaque load. The presence of the mutations also reduced the effect of ADAM10 as a stimulator of adult hippocampal neurogenesis [22].

Third, the mutation segregated consistently with the disease in the family members. Lastly, the biological effect of the p.Tyr167* mutation as a loss of function was supported by a 50% decrease of all ADAM10 isoforms in CSF of mutant cases as compared to controls, as well as a significant reduction of CSF sAPPα—around 70% as compared to controls and sporadic AD cases—which was consistent with a decrease of the α-secretase pathway processing of APP. It is also remarkable that sAPPβ levels in mutants were similar to sporadic AD cases and controls. Previous studies have described a positive correlation between sAPPα and sAPPβ levels in CSF from sporadic AD cases, indicating a similar shift for both fragments [23]. Therefore, only mutant cases displayed a significant decrease of the sAPPα/sAPPβ ratio.

It is interesting that in CSF we found a pattern of reduced ADAM10 mature species in the p.Tyr167* mutation carriers resembling sporadic AD cases. We previously reported that sADAM10 and ADAM10f species were significantly decreased in sporadic AD cases compared to controls [15]. Because of the similarities in clinical features and the ADAM10 biomarker between mutation carriers and sporadic AD cases, we hypothesize that an age-related downregulation of α-secretase activity may be a major mechanism underlying the physiopathogenicity of sporadic AD. In fact, several studies have described decreased α-secretase activity in sporadic AD cases compared to controls when analyzing either ADAM10 or its neurotrophic metabolite sAPPα. There is evidence of an overall decrease of ADAM10 mRNA in brain tissue, a protein decrease in platelets and CSF, and a decrease of sAPPα in platelets and CSF as well [15, 24, 25]. On the contrary, ADAM10 levels in platelets are increased in cognitively preserved octogenarians, suggesting that enhanced α-secretase activity contributes to cognitively healthy aging or confers resilience to neurodegeneration [26]. In this study, despite a similar decrease of ADAM10 levels in CSF obtained from mutants and sporadic AD cases, only the former had a clear impact on APP processing as evidenced in sAPPα levels. We could speculate as to whether a long-term ADAM10 decrease due to a genetic variant has a more pronounced impact on the α-secretase pathway processing of APP compared to a senile age-related downregulation of α-secretase activity. However, the low number of samples with mutation and some dispersion in sAPPα levels in controls and sporadic AD cases prevents us from reaching strong conclusions.

### Limitations

There are some limitations to the study of this family. The family pedigree is small and we could not find a second hit to further support co-segregation. One of the siblings (I.4) had only a brief clinical assessment because he rejected follow-up, though the first clinical evaluation allowed to consider him as affected by incipient dementia. PET neuroimaging with amyloid tracers was not available at our institution, but CSF biomarkers allowed for biological characterization [19] of two affected cases. Regarding basic research, we did not conduct cell or animal-based studies with this particular mutation but the haploinsufficiency effect of the mutation was evidenced by the decrease in CSF ADAM10 and sAPPα levels. Furthermore, a mouse model with a very close *ADAM10* Q170 missense mutation [22] strongly supports the pathogenic effect of *ADAM10* prodomain mutations. Finally, the study of the family excluded the role of variants within the selected gene panel, though the possibility of finding additional rare variants showing a pattern of segregation in a genome-wide search could not be ruled out. However, this possibility does not diminish the impact of the ADAM10 mutation.

## Conclusion

In conclusion, this family represents a new genetic example supporting the amyloid hypothesis in the development of AD and further encourages research into the α-secretase pathway to identify ADAM10 stimulators.

## Data Availability

All data generated or analyzed during this study are included in this published article.
